# Transcriptome reveals the role of the *htpG* gene in mediating antibiotic resistance through cell envelope modulation in *Vibrio mimicus* SCCF01

**DOI:** 10.3389/fmicb.2023.1295065

**Published:** 2024-01-04

**Authors:** Zhenyang Qin, Kun Peng, Yang Feng, Yilin Wang, Bowen Huang, Ziqi Tian, Ping Ouyang, Xiaoli Huang, Defang Chen, Weimin Lai, Yi Geng

**Affiliations:** ^1^College of Veterinary Medicine, Sichuan Agricultural University, Chengdu, Sichuan, China; ^2^Department of Aquaculture, Sichuan Agricultural University, Chengdu, Sichuan, China

**Keywords:** HtpG, *Vibrio mimicus*, transcriptome, drug resistance, cell wall, cell membrane

## Abstract

HtpG, a bacterial homolog of the eukaryotic 90 kDa heat-shock protein (Hsp90), represents the simplest member of the heat shock protein family. While the significance of Hsp90 in fungal and cancer drug resistance has been confirmed, the role of HtpG in bacterial antibiotic resistance remains largely unexplored. This research aims to investigate the impact of the *htpG* gene on antibiotic resistance in *Vibrio mimicus*. Through the creation of *htpG* gene deletion and complementation strains, we have uncovered the essential role of *htpG* in regulating the structural integrity of the bacterial cell envelope. Our transcriptomics analysis demonstrates that the deletion of *htpG* increases the sensitivity of *V. mimicus* to antimicrobial peptides, primarily due to upregulated lipopolysaccharide synthesis, reduced glycerophospholipid content, and weakened efflux pumps activity. Conversely, reduced sensitivity to β-lactam antibiotics in the Δ*htpG* strain results from decreased peptidoglycan synthesis and dysregulated peptidoglycan recycling and regulation. Further exploration of specific pathway components is essential for a comprehensive understanding of *htpG*-mediated resistance mechanisms, aiding in the development of antimicrobial agents. To our knowledge, this is the first effort to explore the relationship between *htpG* and drug resistance in bacteria.

## 1 Introduction

The highly conserved Heat shock proteins (HSP) family, found in both prokaryotic and eukaryotic organisms, rapidly adjusts expression under stress conditions, playing essential roles in protein processes (folding, structural maintenance, and disaggregation) and critical cellular pathways (Backe et al., 2020). They are categorized into six families based on molecular weight, one of which is the 90 kDa heat-shock protein (Hsp90). The high-temperature protein G (HtpG) is a bacterial homolog of the eukaryotic Hsp90 and constitutes a significant proportion of the total protein content (0.36% at 37°C; Mason et al., 1999). Furthermore, HtpG has been identified as a virulence factor across multiple bacterial pathogens (Grudniak et al., 2018; Dong et al., 2021), it plays a crucial role in bacterial resistance to various environmental stresses (Garcia-Descalzo et al., 2011; Honoré et al., 2017), host defense responses, and adaptation to the host environment. Thus, the function of HtpG is critical for survival and dispersal in host and environment habitats of bacterial pathogens. Over the past decades, eukaryotic Hsp90 has emerged as a crucial target for cancer therapy (Lacey and Lacey, 2021) and the treatment of fungal infections (Li et al., 2022; Yin et al., 2022), resulting in numerous ongoing clinical trials evaluating Hsp90 inhibitors (Graner, 2021; Marcyk et al., 2021; Li and Luo, 2022). Cancer research has demonstrated that Hsp90 promotes malignant behaviors of cancer cells (Lacey and Lacey, 2021), such as uncontrolled proliferation, immune evasion, therapy resistance, and so on. Disruption of the chaperone mechanism of Hsp90 represents a potential method to inhibit tumor, as it can enhance the drug sensitivity of cancer cells (Li et al., 2019; Mathieu et al., 2019). Studies in fungi have demonstrated that elevated Hsp90 levels expedite the emergence of resistance to fungicides (Iyer et al., 2022), while reducing Hsp90 activity significantly increases the efficacy of fungicides (Fu et al., 2022; Li et al., 2022). However, it is surprising that there is little literature addressing the contribution of Hsp90 (HtpG) to bacterial drug resistance, particularly in the context of bacterial resistance emerging a global health challenge.

*V. mimicus* is an emerging zoonotic gram-negative bacterial pathogen that can infect a wide range of fish species (Guardiola-Avila et al., 2021), including Siluriformes, Cypriniformes, and Perciformes (Elgendy et al., 2022), as well as crustaceans (Jiang et al., 2022), resulting in severe vibriosis. The disease has rapid onset, rapid progression, and high mortality that can reach 80–100% (Geng et al., 2014). Additionally, *V. mimicus* can endanger human health by contamination food, water, and wounds (Yang et al., 2021), leading to life-threatening cholera-like diarrhea and septicemia. *V. mimicus* shares a remarkably similar genome and ecological niche with *Vibrio cholerae*, displaying adaptability to the human host (Hernandez-Robles et al., 2021), thereby positioning itself as a potential pandemic pathogen (Halder et al., 2022). Antibiotics are currently the most critical method of combating bacterial diseases. However, it has been observed that *V. mimicus* has started exhibiting resistance under the pressure of prolonged clinical antibiotic usage. A wealth of global evidence suggests its continuous evolution or acquisition of resistance to various drugs, notably β-lactam antibiotics such as ampicillin, amoxicillin, penicillin G, and carbenicillin (Beshiru et al., 2020; Karen Alvarez-Contreras et al., 2021; Elgendy et al., 2022), alongside polymyxin B, azithromycin (Gxalo et al., 2021), sulfamethoxazole, trimethoprim (Adesiyan et al., 2021), and doxycycline (Adesiyan et al., 2021). Given the potential risk of significant outbreaks, the challenges associated with treating infections, and the emerging trend of drug resistance, it becomes imperative to initiate research into the mechanisms of drug resistance in *V. mimicus* at an early stage.

In this study, we assessed changes in drug resistance resulting from *htpG* gene deletion in *V. mimicus* and characterized the global transcriptome changes pre- and post-gene deletion. By integrating phenotypic assessments with variations gene expression variations, we aimed to uncover the underlying mechanisms through which bacterial *htpG* contributes to drug resistance.

## 2 Materials and methods

### 2.1 Bacterial strains and culture conditions

The bacterial strains and plasmids used in this study are listed in Table 1. The strains were cultured in the Luria-Bertani (LB) medium at 28, 37, or 42°C. In Δ*htpG*/p*htpG* Strain, synthesis of HtpG was induced by addition of final concentrations 100 μg/mL L-arabinose (Solarbio, L8060, Beijing, China) at time of culture.

**Table 1:** Bacterial strains and plasmids used in this study.

**Strain or plasmid**	**Description**	**Reference or source**
***V. mimicus***		
SCCF01	Pathogenic wild-type (WT)	Yu et al., 2020
Δ*htpG*	WT with a deletion in *htpG* gene	This study
Δ*htpG*/p*htpG*	Δ*htpG* with complement plasmid contain *htpG* gene	This study
***Escherichia coli***		
DH5α	Competent cells for plasmid cloning	Tsingke Biotech Co., Ltd
**Plasmid**		
pKD3	Plasmid with FRT-flanked chloramphenicol-resistance gene	Miaolingbio Co., Ltd
pCP20	Temperature-sensitive plasmids, introduce FLP recombinase, Amp^r^	Miaolingbio Co., Ltd
pBAD24	Inducible expression plasmid, Amp^r^	Biofeng Biotech Co., Ltd

### 2.2 Construction of the *htpG* deletion strain and complemented strain

Natural transformation of *V. mimicus* (Yu et al., 2019) was employed to create the *htpG* deletion mutant strain. Briefly, the process involved connecting the upstream and downstream homologous arms of the target gene with the chloramphenicol-resistant gene from the plasmid pKD3 by fusion PCR (specific primers are in Table 2). The fused PCR fragment was introduced into the WT strain by natural transformation. Colonies containing the correct gene deletions were subsequently transformed with the FLP recombinase plasmid pCP20 to remove the chloromycetin resistance marker, and the pCP20 was then cured from the resulting strain by continuous passage at 42°C. Finally, the mutant strain was selected for PCR and sequencing to verify the accurate deletion and genomic location of the target gene (specific primers are in Table 2). Colony with no *htpG* gene and pCP20 detected were used as the *htpG* deletion strain Δ*htpG*. The wild-type *htpG* gene was cloned in a pBAD24 expression vector with the arabinose promoter, then electro-transformed into Δ*htpG* competent cells as the complemented strain Δ*htpG*/*phtpG*. Furthermore, expression levels of *htpG* gene were analyzed by RT-qPCR (primers see VM_10215 gene in Supplementary Table 1).

**Table 2:** Primers used in gene deletion and PCR identification.

**Primers name**	**Primers sequence 5^′^to 3^′^**	**Product length (bp)**	**Primers' role**
UP	(F) TCTGGTGGTTGCTGGCCTCT	2,974	Targeting fragments preparation
	R: cgaagcagctccagcctacaACTTTTGTCGATTCTACTAA			
DN	(F) ggaccatggctaattcccatAATCGCTCCGTTTACTTCTG	3,311		
	R: AAACGGCAGGTGGATGGGCG			
CM	(F) TTAGTAGAATCGACAAAAGTtgtaggctggagctgcttcg	1,072		
	R: CAGAAGTAAACGGAGCGATTatgggaattagccatggtcc			
UD-TC	(F) GATTAAACGTTGTGCTTGAGGT	2,116(Wild-type *htpG*), 1,180(*htpG* replaced by a chloramphenicol resistance cassette), 250 (*htpG* deletion)	Strains genetype detection
	R: TCCATATCTCACTTGCACATCA		
HTPG	(F) ACCACCAATAAAGAAACTCG	1,864	The *htpG* gene near-full-length sequences detection
	R: CCGCACTCAAGAATTGTGAC		

### 2.3 Growth curves and biochemical characterization

Bacterial strains were cultured for 18 h before the start of the experiments and were then diluted to an optical density (OD_600_) of one. Subsequently, the cultures were diluted 1:100 into LB medium and maintained at 28°C with shaking at 180 r/min. We recorded OD_600_ readings at 60-min intervals over a 24-h period. Biochemical analyses were conducted using the automated identification system Vitek^®^ 2 GN ID cards (bioMerieux, France). Each experiment included three parallel samples, and statistical analysis was performed using GraphPad Prism 9.0 (GraphPad Software, San Diego, CA).

### 2.4 Antibiotic susceptibility testing

We performed antimicrobial susceptibility testing for 30 antibiotics using the disk diffusion method recommended by aquatic animal clinical and CLSI guidelines. The antibiotic disks were obtained from Hangzhou Microbial Reagent Co., Ltd (Hangzhou, China). The various drugs and their targets used in this study are summarized in Supplementary Table 2, covers 12 different classes of antibiotics and related compounds, namely: penicillins, carbapenems, cephalosporins, aminoglycosides, macrolides, polypeptides, sulfonamides, quinolones, rifamycins, chloramphenicol, tetracyclines, and nitrofuran. Each strain underwent triplicate testing with a single drug, and the mean value was used to determine its diameter of the bacteriostasis circle. Data analysis and visualization were performed using R software packages, including ggplot2 (Version 3.4.0), aplot (Version 0.1.9), and ggtree (Version 3.6.2). Subsequently, the normalized diameter of the bacteriostasis circle was used to generate the heatmap.

### 2.5 Measurement of cell envelope permeability

Cell membrane and cell wall permeability were assessed using propidium iodide (PI) and alkaline phosphatase (ALP) assay kits. The bacteria were cultured for 8 h to reach the mid-logarithmic phase. Subsequently, they were washed in 100 mM PBS (pH 7.3), and the cell density of the bacterial suspensions was adjusted to an OD_600_ of 0.4–0.5, ensuring uniform cell counts for each bacterial strain. Then, PI (Solarbio, C0080) was added to a final concentration of 10 mM and incubated at 28°C for 30 min. The fluorescence value was measured using a fluorescence plate reader (Thermo, Varioskan Flash) with excitation wavelength at 535 nm and an emission wavelength at 615 nm. The bacterial culture supernatants were collected and centrifuged at 12,000 r/min for 10 min. The ALP activity detection assay was performed using the AKP/ALP detection kit (Solarbio, BC2140) following the manufacturer's instructions. The measurements were performed in triplicate, and the data were presented as mean ± SE. Significance (*p* < 0.05) was determined using one-way ANOVA.

### 2.6 Transmission electron microscopy (TEM) analysis of the cell envelope

After being grown on LB agar at 28°C for 18 h, the colony was fixed overnight in 0.1 M sodium phosphate buffer (pH 7.4) containing 3% glutaraldehyde at 4°C. Samples were fixed in 0.1 M sodium phosphate buffer (pH 7.4) containing 1% OsO_4_ for 2 h. Subsequently, they were sequentially dehydrated in 50, 70, 80, 90, 95, and 100% ethanol, and finally in 100% acetone, with each dehydration step lasting for 15 min. The samples were embedded in 812 epoxy resin monomer (SPI) and then sliced into ultrathin sections measuring 60–80 nm using a Leica UC7 ultrathin microtome. These sections were subsequently stained with uranyl acetate and lead citrate before being imaged at 80 kV using a JEOL JEM-1400FLASH transmission electron microscope. The assessment of cell wall structure integrity involved calculating the percentage of cells with abnormal structures from images taken at a 12,000× magnification. Cells with intact and well-defined cell wall structures were considered normal, while those displaying cell wall undulating folds or rupture, or expanded periplasmic space were categorized as having abnormal structures.

### 2.7 RNA extraction and sequencing

The 18-h cultures of *V. mimicus* SCCF01 and Δ*htpG* strains were diluted to an OD_600_ of 1 before initiating the experiments. Subsequently, cultures were diluted 1:100 into 5 mL LB medium and incubated at 28°C with shaking for 12 h. Bacterial cultures were centrifuged at 12,000 r/min for 5 min. Total RNA was extracted from the bacterial precipitate using RNAprep Pure Cell/Bacteria Kit (TIANGEN, Beijing, China) following the manufacturer's instructions and genomic DNA was removed using DNase I (TaKaRa). Then RNA quality was determined by 2100 Bioanalyser (Agilent) and was checked by RNase-free agarose gel electrophoresis, and quantified using the ND-2000 (NanoDrop Technologies). Only high-quality RNA sample (OD_260/280_ = 1.8–2.2, OD_260/230_ ≥ 2.0, RIN ≥ 6.5, 28S:18S ≥ 1.0, >10 μg) was used to construct sequencing library.

The transcriptome library was prepared using the TruSeq^TM^ Stranded Total RNA Library Prep Kit (Illumina, San Diego, CA). Ribosomal RNA (rRNA) depletion was performed using the Ribo-Zero Magnetic kit from Epicenter, and then fragmented using a fragmentation buffer. Subsequently, double-stranded cDNA was synthesized using the SuperScript double-stranded cDNA synthesis kit (Invitrogen, CA) with random hexamer primers (Illumina, San Diego, CA). The synthesized cDNA underwent end-repair, phosphorylation, and “A” base addition following Illumina's library construction protocol. Subsequently, the second-strand cDNA was digested using UNG (Uracil-N-Glycosylase). The cDNA fragments were then size-selected through agarose gel electrophoresis, PCR amplified, and sequenced using the Illumina HiSeq^TM^ X Ten platform with a read length of 2×150 bp. This study included three biological replicates.

### 2.8 Transcriptomic analysis

The fastp software^1^ (Version 0.20.1) was used to clean and evaluate the qualities of the raw reads from sequencing. The reads containing the adapter, poly-N, and more than 40% low-quality bases (Q < 20) were cleaned. The cleaned reads were mapped onto the genome of the *V. mimicus* SCCF01 strain (GenBank accession no. GCA_001767355) as the reference genome using bowtie2 (Version 2.2.9). Gene Ontology (GO) and Kyoto Encyclopedia of Genes and Genomes (KEGG) pathway annotation and enrichment analyses were based on the GO Database^2^ and KEGG pathway database.^3^ Gene expression levels were calculated and normalized as TPM approach using by RSEM^4^ (Version 1.3.1). The differential gene expression was analyzed using the DESeq2 package^5^ (Version 1.24.0), in which the *p* was adjusted by the Benjamini-Hochberg (BH) method, belonging to the False Discovery Rate (FDR) for correction. The genes with the adjusted *p* < 0.05 and the absolute value of log_2_ (FC) > 1 were deemed as the DEGs. The GO enrichment and KEGG enrichment were conducted using the clusterProfiler package (Guangchuang et al., 2012) to identify the biological functions and pathways mainly affected by the DEGs. Analyses not mentioned above were conducted using the online platform of Majorbio Cloud Platform^6^ (Ren et al., 2022).

### 2.9 Validation of RNA sequencing

To validate the results obtained from the transcriptome, we selected 10 upregulated genes and 11 downregulated genes of interest for two-step RT-qPCR verification with the same RNA-seq samples. Reverse transcription was performed using the *Evo M-MLV* RT Master Mix for qPCR kit (AG11706, Accurate Biology, China), and real-time qPCR was carried out using the SYBR^®^ Green *Pro Taq* HS Premix kit (AG11701, Accurate Biology, China). The Bio-Rad CFX96 qPCR System was used for RT-qPCR, and using the reaction as follows: initial denaturation at 95°C for 30 s, followed by 40 cycles of reaction at 95°C for 5 s and 60°C for 30 s. The melting curve was generated at the end of the cycle to confirm the specificity of the amplification product. Each PCR reaction was repeated three times. Gene expression was calculated and compared using the 2^−ΔΔCt^ method, with *16s rRNA* serving as an endogenous reference. The primers used for the experiment are listed in Supplementary Table 1.

## 3 Results

### 3.1 Deletion and complementation of *htpG* exhibit no effect on bacterial growth and principal biochemical phenotype

**Figure 1:**
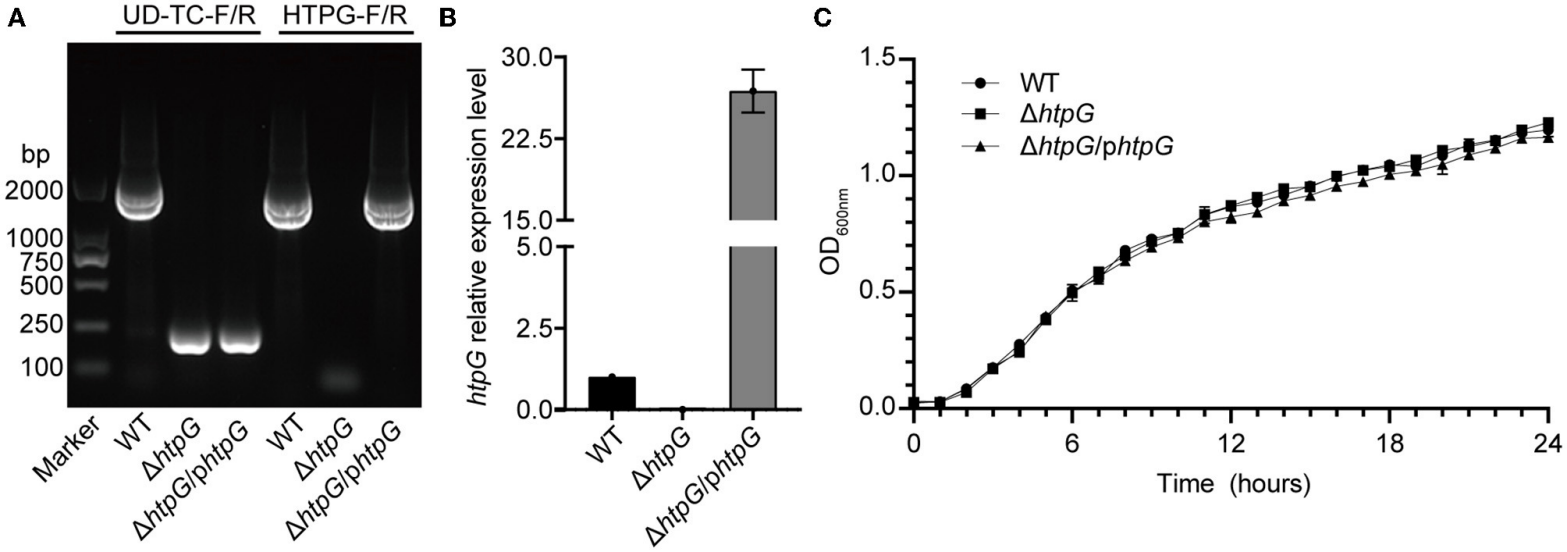
Identification of *htpG* gene deletion and complementation strains. **(A)** Electrophoretogram displaying the results of strains validation by PCR (the UD-TC-F/R primers were used to confirm gene knockout from the specified genomic position, while the HTPG-F/R primers were used to identify the presence of the target gene within the bacterial strain). **(B)** Relative expression level of the *htpG* gene among the test strains determined by RT-qPCR. **(C)** Growth curves of the tested strains under 28°C with shaking.

PCR and RT-qPCR were employed to verify the successful construction of the *htpG* deletion and complementation strain. Agarose gel eletrophoresis of PCR products revealed that the fragments were shorter when using the primer pair UD-TC-F/R in Δ*htpG* and Δ*htpG/phtpG*, indicating the deletion of *htpG* in the bacterial genome. With the primer pair HTPG-F/R, fragments of the *htpG* gene size (1,864 bp) were present in Δ*htpG*/p*htpG* and the WT strain (Figure 1A). All strains were verified by sequencing, confirming the successful construction of *htpG* gene deletion and complementation strains. Expression of *htpG* was significantly higher in complemented strains than in parent strains (Figure 1B). Furthermore, these three strains exhibited similar growth curves (Figure 1C), indicating that the deletion and overexpression complementation of *htpG* do not impact the growth of *V. mimicus* under experimental conditions. In the biochemical characterization test of these strains, only six out of 47 indicators showed inconsistencies, namely Tyrosine arylaminases (TyrA), succinate alkalinization (SUCT), phosphatase (PHOS), ornithine decarboxylase (ODC), coumarate (CMT), and Glu-Gly-Arg-arylamidase (GGAA). However, these differences did not impact the high confidence of the identification results, as shown in Supplementary Table 3. This suggests that *htpG* gene deletion and complementation do not significantly affect the principal biochemical phenotype of *V. mimicus*.

### 3.2 The deletion of *htpG* affects *V. mimicus* antibiotic resistance targeting the cell envelope

**Figure 2:**
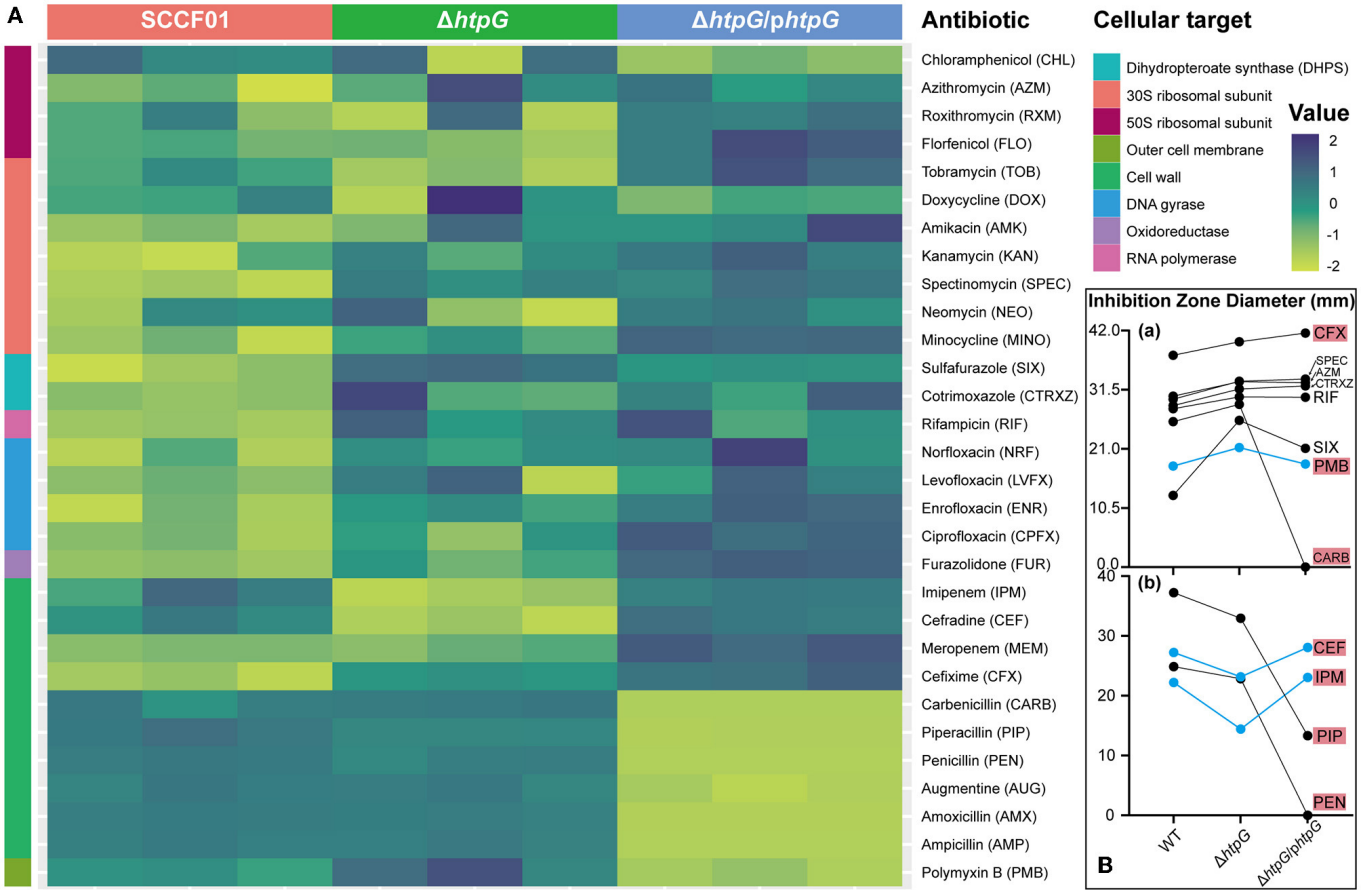
Impact of *V. mimicus htpG* gene deletion and complementation on sensitivity to thirty antibiotics. **(A)** Heatmap of antibiotic resistance of the tested bacterial strains based on normalized inhibition zone diameters (susceptibility results are categorized by cellular targets. Dark blue represents the maximum diameter of the inhibition zone, while light green represents the minimum diameter. The transition from light green to dark blue indicates a gradual increase in the inhibition zone diameter). **(B)** Changes in the inhibition zone diameters for twelve antibiotics exhibiting significant sensitivity alterations. (a) Eight antibiotics with upregulated drug sensitivity. (b) Four antibiotics with downregulated drug sensitivity (blue lines represent restored resistance phenotypes in the complemented strains. Abbreviations on the right side indicate the corresponding antibiotics, with a red background denoting antibiotics targeting the cell membrane or cell wall).

By utilizing a normalized heatmap (Figure 2A), we observed a generally consistent trend in the resistance changes of *htpG* gene deletion and complemented strains to a wide range of antibiotics targeting the same mechanism. The sensitivity of the Δ*htpG* to cefixime (CFX), spectinomycin (SPEC), cotrimoxazole (CTRXZ), azithromycin (AZM), rifampicin (RIF), sulfafurazole (SIX), Polymyxin B (PMB), and carbenicillin (CARB) was significantly increased (difference value > 2 mm; Figure 2B-a), while the sensitivity to cefradine (CEF), imipenem (IPM), piperacillin (PIP), and penicillin (PEN) was significantly decreased (difference value > 2 mm; Figure 2B-b). Besides, the resistance of the Δ*htpG*/p*htpG* to PMB, CEF, and IPM could be restored to WT strain level (Figure 2B). The Δ*htpG/phtpG* strain maintained resistance level to norfloxacin (NRF), levofloxacin (LVFX), ciprofloxacin (CPFX), enrofloxacin (ENR), RIF, AZM, CTRXZ, SPEC, and CFX, indicating that the complementation of the *htpG* gene could not restore resistance changes to these antibiotics. In addition, resistance to doxycycline (DOX), neomycin (NEO), amikacin (AMK), and kanamycin (KAN) in *V. mimicus* was not affected by either *htpG* deletion or complementation, indicating that *htpG* does not mediate the regulation of resistance to these four antibiotics. Due to the presence of the AMP resistance gene on the pBAD24 plasmid, the diameter of the bacteriostasis zone for the Δ*htpG/phtpG* strain against certain penicillin drugs, namely CARB, PEN, amoxicillin (AMX), and ampicillin (AMP) was 0 mm, and there was an increased resistance to augmentine (AUG) and piperacillin (PIP). Among the 12 drugs with significantly altered sensitivity, seven of them target the cell wall or cell membrane.

### 3.3 The *htpG* maintains the permeability and structural integrity of the cell envelope

**Figure 3:**
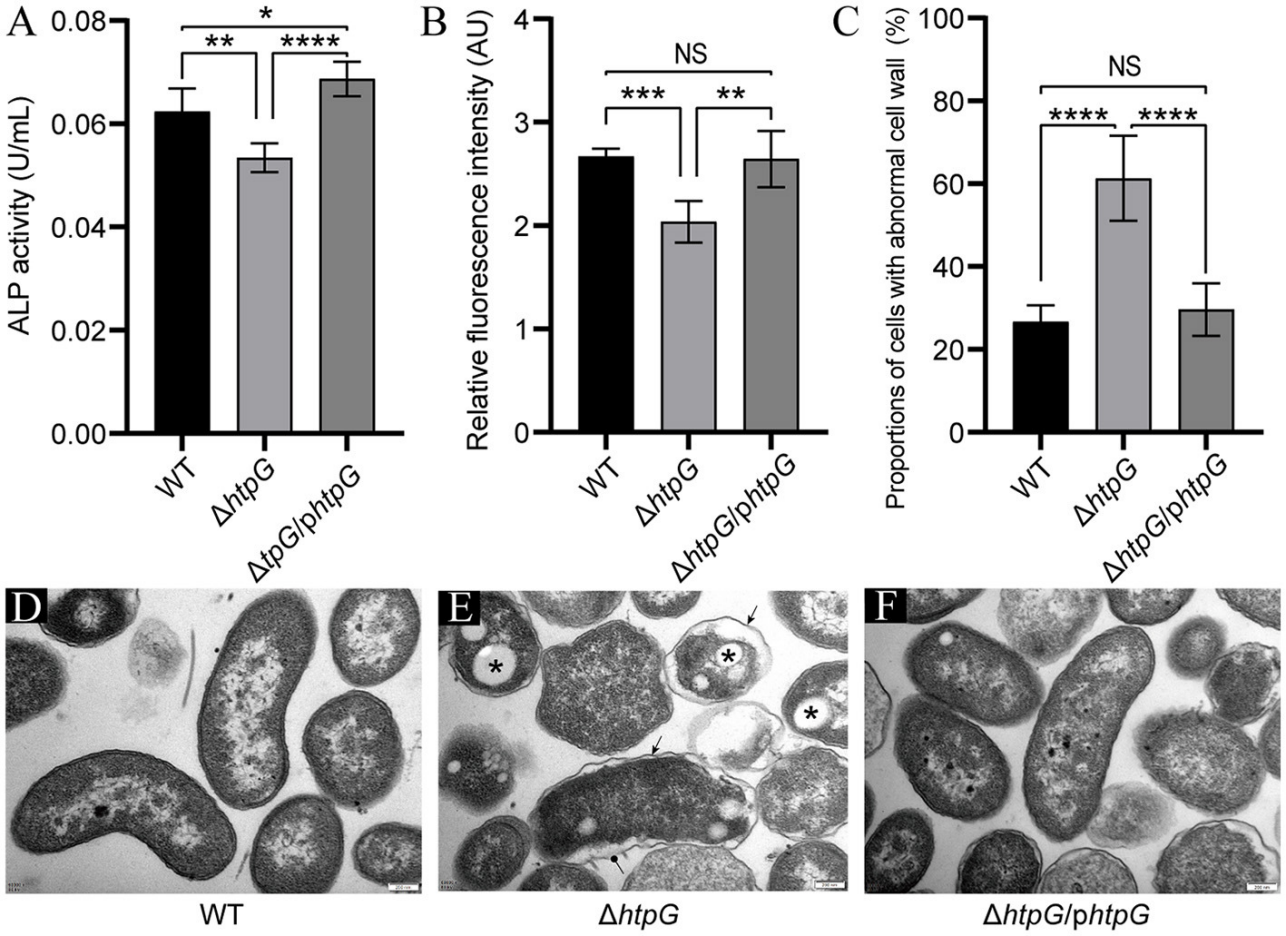
Impact of *htpG* deletion and complementation on cell wall and cell membrane permeability and integrity. **(A)** Alkaline phosphatase (ALP) activity in culture supernatant. **(B)** Relative propyl iodide (PI) fluorescence intensity in collected bacterial cells. **(C)** Quantification of the proportion of cells exhibiting abnormal cell wall structures based on transmission electron microscopy (TEM) images captured at a magnification of 12,000×. **(D–F)** TEM images of *V. mimicus* WT **(D)**, Δ*htpG* **(E)**, and Δ*htpG*/p*htpG* **(F)** strains [scale bar = 200 μm; sharp arrows in **(E)** point to cell wall undulating folds, circular solid arrows point to ruptured cell walls, and asterisks denote presumed accumulation of Poly-3-d-hydroxybutyrate (PHB)].

To confirm the impact of *htpG* deletion and complementation on cell envelope, we assessed the permeability and integrity of the cell membrane and cell wall. Deletion of *htpG* resulted in a significant reduction in both alkaline phosphatase (ALP) levels and propyl iodide (PI; *p* < 0.01). Meanwhile, the complemented strain restored this phenotype (Figures 3A, B), indicating a positive correlation between *htpG* and cell wall and cell membrane permeability. On the other hand, TEM images showed intact and well-defined cell wall and membrane in the majority of the WT strain cells (Figure 3D). Compared with the WT strain, the Δ*htpG* strain cell wall structure was disordered, the distance between the cell membrane and the cell wall widened, and the cell shrank slightly; clearer, rounded, and well-demarcated structures were observed in the cytoplasm (Figure 3E). Complemented strain cells' morphology became closer to WT cells (Figure 3F). The statistics revealed that the proportion of cells with abnormal cell wall structures significantly increased from 26.68 ± 3.73% to 61.27 ± 9.67% (*p* < 0.0001) following *htpG* gene deletion. However, this proportion decreased to 29.60 ± 5.98% after gene product complementation (Figure 3C). Therefore, *htpG* plays a role in maintaining the structural integrity of the cell envelope in *V. mimicus*.

### 3.4 Transcriptome analysis reveals the global regulatory functions of *htpG* in *V. mimicus*

**Figure 4:**
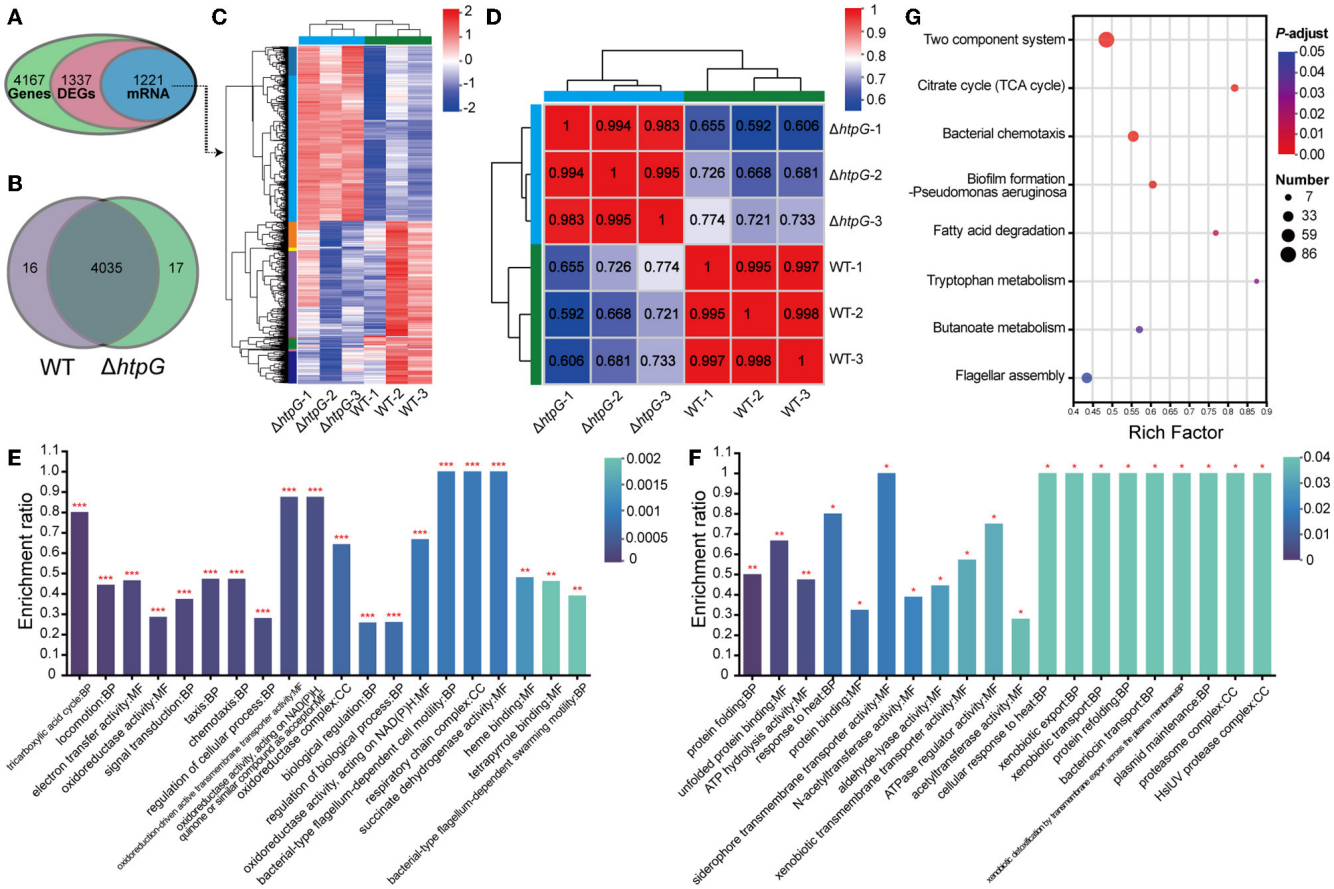
Overview of the transcriptome profiles of *V. mimicus* WT and Δ*htpG* strains. **(A)** The number of genes obtained for analysis after transcriptome data quality control, quantification, and filtering. **(B)** The number of genes with expression levels TPM > 1 that are unique or shared between the two bacterial strains. **(C)** Heat map overview of the expression levels of 1,221 differentially expressed mRNA in the two bacterial strains. Higher expression is represented in red, while lower expression is represented in blue. **(D)** Heat map depicting the expression correlation among samples from the two groups. The numbers indicate the correlation coefficients between corresponding samples; closer values to 1 are shown in red, and lower values are displayed in blue. **(E, F)** GO enrichment analysis results of 636 up-regulated mRNAs **(E)** and 585 down-regulated mRNAs **(F)**. The top 20 enriched terms are presented. The vertical axis represents the enrichment ratio, where higher values indicate greater enrichment. The color signifies the significance of enrichment, with *** denoting FDR < 0.001, ** for FDR < 0.01, and * for FDR < 0.05. **(G)** KEGG pathway enrichment analysis of the 1,221 differentially expressed mRNA. The x-axis represents the Rich Factor, with larger values indicating higher enrichment levels. The size of the points reflects the number of genes in the pathway, and the color of the points represents the *p*-adjust value.

To further investigate the impact of *htpG* deletion on the overall gene expression in *V. mimicus* and elucidate the mechanisms governing its effects on cell membrane permeability and integrity, we conducted transcriptomic analysis. The transcriptome analysis of the WT strain SCCF01 and the deletion strain Δ*htpG* detected a total of 4,167 genes, out of which 1,337 were identified as differentially expressed genes (DEGs, fold changes≥2 or ≤ 0.5, *p* ≤ 0.05, detailed information see in Supplementary Table 4), including 1,221 mRNAs (636 upregulated and 585 downregulated; Figures 4A, C, Supplementary Figure 1A). Venn analysis revealed that out of 4,068 genes with TPM > 1, 17 were unique to the WT group, while 16 were unique to the Δ*htpG* group (Figure 4B). Correlation analysis of samples demonstrated higher correlations within groups compared to those between groups, indicating robust repeatability within each group (Figure 4D, Supplementary Figure 1B). GO enrichment analysis (Supplementary Table 5) revealed up-regulated mRNAs were associated with cellular energy metabolism, process regulation, and bacterial locomotion (Figure 4E), while down-regulated mRNAs were linked to metabolic regulation, stress adaptation, and substance transport (Figure 4F). Furthermore, all DEGs were mapped to 166 KEGG pathways (Supplementary Table 6). The enriched KEGG pathways included pivotal processes such as the two-component system, bacterial chemotaxis, flagellar assembly, and pyruvate metabolism (Figure 4G). A significant number of DEGs were implicated in energy source and amino acid biosynthesis and metabolism, exemplified by pathways such as the citrate cycle (TCA cycle), fatty acid degradation, butanoate metabolism, tryptophan metabolism, and valine, leucine, and isoleucine degradation.

### 3.5 The deletion of *htpG* alters cell membrane components expression, including lipopolysaccharides, glycerophospholipids, and membrane proteins

**Figure 5:**
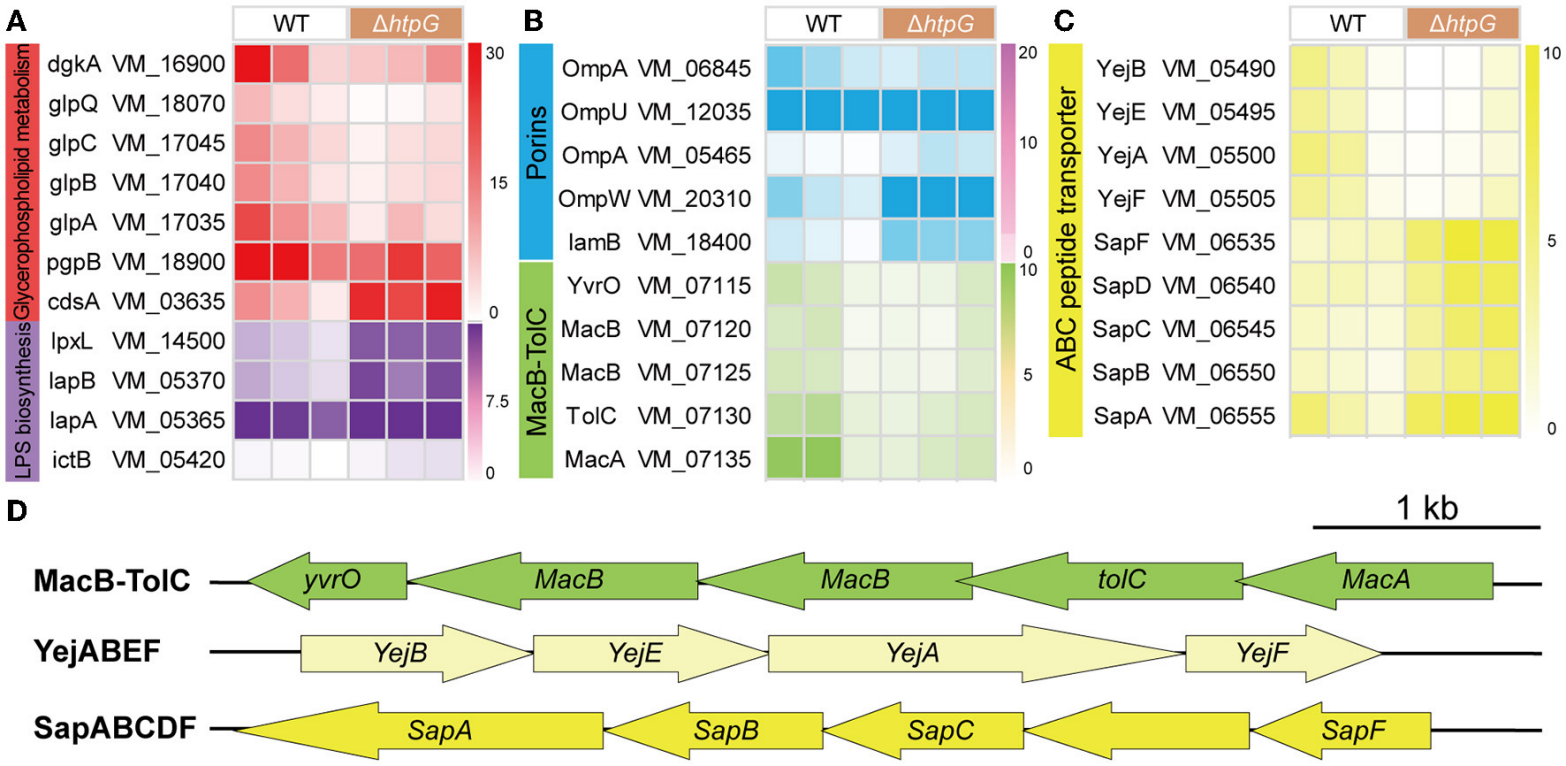
The impact of *htpG* deletion on the gene expression of cell membrane components. **(A–C)** Heat map of gene expression related to cellular membrane components. **(A)** Lipopolysaccharide (LPS) biosynthesis and glycerophospholipid metabolism, **(B)** Porin and Mac-TolC efflux pumps, **(C)** ABC peptide transporter. **(D)** The operon structure of Mac-TolC efflux pump and ABC peptide transporter gene cluster of *V. mimicus*. Scale bar = 1,000 bp.

Further, we conducted a detailed analysis of the significant gene expression changes affecting various key components of the cell envelope to elucidate the specific impact of *htpG*. Initially, we examined genes related to cellular membrane components, which are also associated with antimicrobial peptide resistance. In the intricate biosynthesis and assembly pathway of Lipopolysaccharide (LPS), notable upregulation was observed in VM_14500 (lpxL), VM_05370 (*lapB*), VM_05365 (*lapA*), and VM_05420 (*ictB*) (Figure 5A). These genes are critical for processes such as lipid A biosynthesis, O-antigen linkage, and LPS assembly. However, no significant impact was detected on other genes within this pathway. Including VM_16900 (*dgkA*), VM_18070 (*glpQ*), VM_18900 (*pgpB*), VM_17045 (*glpC*), VM_17040 (*glpB*), and VM_17035 (*glpA*), the key enzymes in the synthesis pathways of the major intracellular membrane constituents, Phosphatidylethanolamine (PE) and phosphatidylglycerol (PG), exhibited significant downregulation, with only VM_03635 (*cdsA*) showing a significant upregulation (Figure 5A). These findings suggest that the deletion of *htpG* promotes the synthesis of LPS while inhibiting glycerophospholipid biosynthesis. Regarding drug resistance-associated porins, VM_05465 (*ompA*), VM_20310 (*ompW*), and VM_18400 (*lamB*) were upregulated, while VM_06845 (*ompA*) and VM_12035 (*ompU*) were downregulated (Figure 5B). Notably, the porin genes associated with drug resistance exhibited an overall upregulation in terms of fold change in expression.

As for the ATP-binding cassette transporter (ABC) family proteins involved in nutrient uptake, toxin secretion, and antibiotic efflux for bacterial resistance, 12 DEGs exhibited significant variation. These DEGs can be classified into three operons associated with drug resistance (Figure 5D). The complete *MacAB-TolC* operon, including VM_07115 (*yvrO*), VM_07120 (*macB*), VM_07125 (*macB*), VM_07130 (*tolC*), and VM_07135 (*macA*), was significantly downregulated (Figure 5B). The VM_05500 (*yejA*), VM_05490 (*yejB*), VM_05495 (*yejE*), and VM_05505 (*yejF*) which belong to the *YejABEF* operon, were significantly down-regulated (Figure 5C). Conversely, within the *SapABCDF* operon, VM_06550 (*sapB*), VM_06545 (*sapC*), and VM_06535 (*sapF*) exhibited significant upregulation, whereas VM_06540 (*sapD*) and VM_06555 (*sapA*), also part of this operon, showed elevated expression (though not significant; Figure 5C).

### 3.6 The deletion of *htpG* causes profound peptidoglycan metabolism disruption

**Figure 6:**
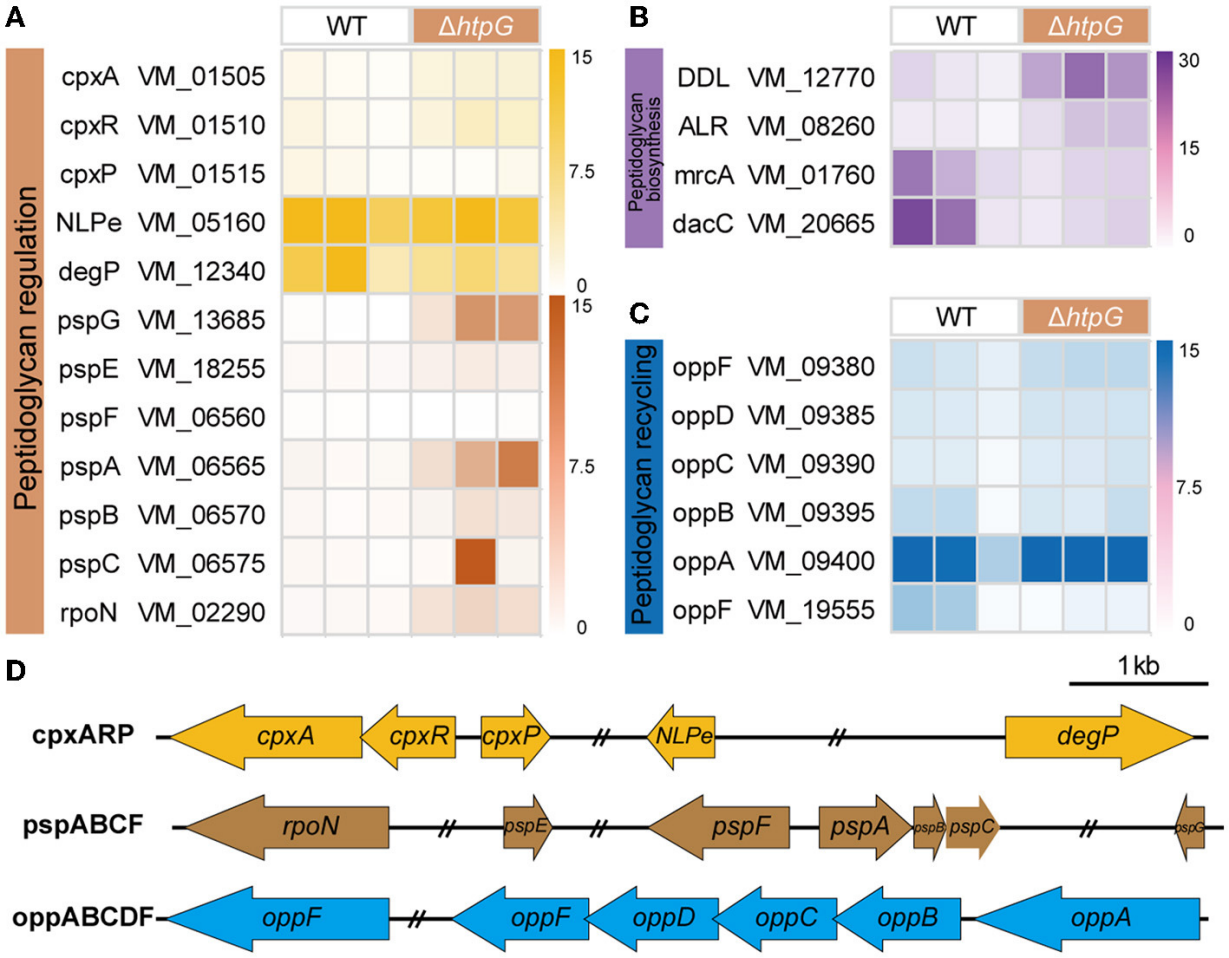
The effect of *htpG* deletion on the expression of peptidoglycan metabolism-related genes. **(A–C)** Heat map of gene expression related to peptidoglycan metabolism. **(A)** Peptidoglycan regulation, **(B)** peptidoglycan biosynthesis, and **(C)** peptidoglycan recycling. **(D)** The operon structure of the peptidoglycan regulation and recycling gene cluster of *V. mimicus*. Scale bar = 1,000 bp, double slash marks indicate the presence of sequence gaps.

Subsequently, our analysis focused on genes related to the major component of the cell wall, peptidoglycan, including biosynthesis, recycling, and regulation. These components are pivotal in peptidoglycan metabolism and serve as essential and well-established targets for β-lactam antibiotics (Kang and Boll, 2022). In the Peptidoglycan biosynthesis pathway, two DEGs located at its initiation, VM_12770 (D-alanyl-D-alanine ligase, DDL), and VM_08260 (alanine racemase, ALR), exhibited significant upregulation. Conversely, at terminal end of the pathway, two DEGs belonging to the penicillin-binding proteins (PBPs), VM_01760 (mrcA/PBP1a) and VM_20665 (dacC/PBP5), responsible for regulating peptidoglycan layer synthesis and assembly, displayed significant downregulation (Figure 6B). This downregulation markedly impacts cell wall peptidoglycan production and structure.

The bacterial oligopeptide permeases (opp) operon belongs to the ABC superfamilies and is involved in cell wall metabolism by recycling peptidoglycan and peptide into the cytoplasm (Singh et al., 2020). In the *V. mimicus* SCCF01 strain, the entire *oppABCDF* operon was annotated (Figure 6D), and following *htpG* deletion, the substrate-binding protein OppA (VM_09400) exhibited significantly upregulation, whereas the ATP-binding protein OppF (VM_19555) showed significant downregulation (Figure 6C). This indicates an enhanced *oppABCDF* complex affinity for cell wall peptides and peptidoglycan, but the systemic energy supply is insufficient. Bacteria sense and ensure the stability of the cell envelope by utilizing the two-component CpxA/CpxR system and phage shock protein (psp) system. Within the *V. mimicus* SCCF01 strain, a complete *cpxARP* operon has been annotated. Upon *htpG* deletion, VM_01505 (*cpxA*, pressure receptor) and VM_01510 (*cpxR*, response regulator) are significantly upregulated, while VM_01515 (*cpxP*, stress adaptor protein) shows significant downregulation (Figure 6A). This indicates the activation of the cpx system. However, the key activator NlpE (VM_05160) of this complex, and downstream effector protein DegP (VM_12340) were downregulated, although not significantly (Figure 6A). This implies that the optimal activation of the cpx system might not have been achieved or that other unexplored response pathways could be involved. In addition, a classical psp system was annotated, consisting of a *pspFABC* gene cluster and the more distantly located *pspG* (VM_13685) and *pspE* (VM_18255) genes (Figure 6D). Among them, only VM_06565 (*pspA*) and VM_13685 (*pspG*) exhibit significant upregulation. Simultaneously, VM_02290 (*rpoN*), which transcribes the operon along with pspF, also shows significant upregulation (Figure 6A). This highlights the activation of the psp system, which contributes to the stabilization of both the cell membrane and the peptidoglycan layer.

### 3.7 Verification by RT-qPCR

**Figure 7:**
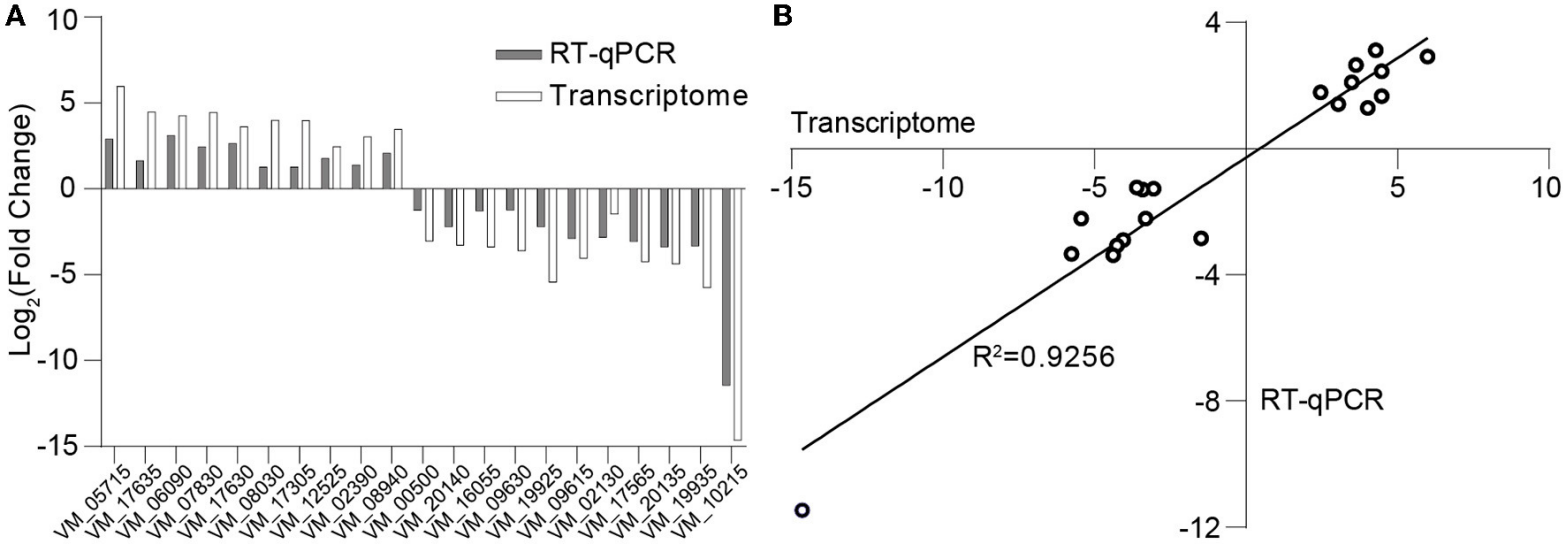
Validation of transcriptome data through RT-qPCR. **(A)** Log-transformed expression fold change achieved via RT-qPCR and RNA-seq for the selected genes. **(B)** Correlation between the differential expression ratio (log_2_) determined by RT-qPCR and RNA-seq for the selected differentially expressed genes.

The differential log-transformed expression fold change of selected DEGs obtained by RT-qPCR and RNA-seq is depicted in the Figure 7A. The gene expression trends observed in RT-qPCR are similar to those in RNA-seq, with a correlation coefficient of *R*^2^ = 0.9256, indicating a strong correlation between the two transcriptions (Figure 7B). The inconsistency may result from variations in sensitivity and procedures between transcriptome sequencing and RT-qPCR.

## 4 Discussion

This study demonstrated that the most significant alterations in antibiotic resistance, primarily targeting the cell envelope, result from the deletion of *htpG*, which compromises the permeability and integrity of the cell envelope. Subsequent transcriptomic analysis of specific genes revealed that *htpG* promotes resistance to antimicrobial peptides by regulating LPS and glycerophospholipid synthesis on the cell membrane, as well as the functionality of MacAB-TolC and YejABEF pumps. Furthermore, it was confirmed that *htpG* mediates the synthesis, recycling, and regulation of peptidoglycans on the cell wall, thereby contributing to increased sensitivity of the strain to β-lactam antibiotics. This highlights the intricate and widespread engagement of *htpG* in bacterial physiological processes, motivating us to further explore a nuanced comprehension of how *htpG* precisely facilitates or hinders resistance to specific categories of drugs. The necessity for further specific experimental research on the regulation of the *htpG* gene in bacterial drug resistance pathways arises from several factors. Firstly, given the potential pandemic risk associated with *V. mimicus* and the escalating trend of antibiotic resistance, it is paramount to initiate investigations into its resistance mechanisms promptly. This research not only facilitates early interventions, including drug guidance, but also contributes to the identification of crucial biomarkers for monitoring. Additionally, the drug resistance mechanisms unveiled in this study have a nearly universal presence in Gram-negative bacteria, and a detailed exploration of their interplay offers new targets for combating drug-resistant pathogens. Furthermore, our study highlights the pivotal role played by the *htpG* gene in regulating sensitivity to critical antibiotics, such as polymyxin B, and imipenem, which are of substantial clinical significance. Imipenem, in particular, serves as a highly recommended frontline defense against severe infections caused by multidrug-resistant bacteria, while polymyxin B remains a global last-resort option. Furthermore, the existence of inhibitors designed for eukaryotic Hsp90 proteins highlights the potential for repurposing these agents to develop safe and effective tools for suppressing bacterial drug resistance. Lastly, previous studies have highlighted that these inhibitors also possess the potential to suppress bacterial virulence (Wickner et al., 2021). Thus, an in-depth exploration of the regulatory networks governing these specific genes promises for identifying future therapeutic targets and improve susceptibility to antimicrobial peptides and β-lactam antibiotics, critical steps in advancing antimicrobial therapy.

**Figure 8:**
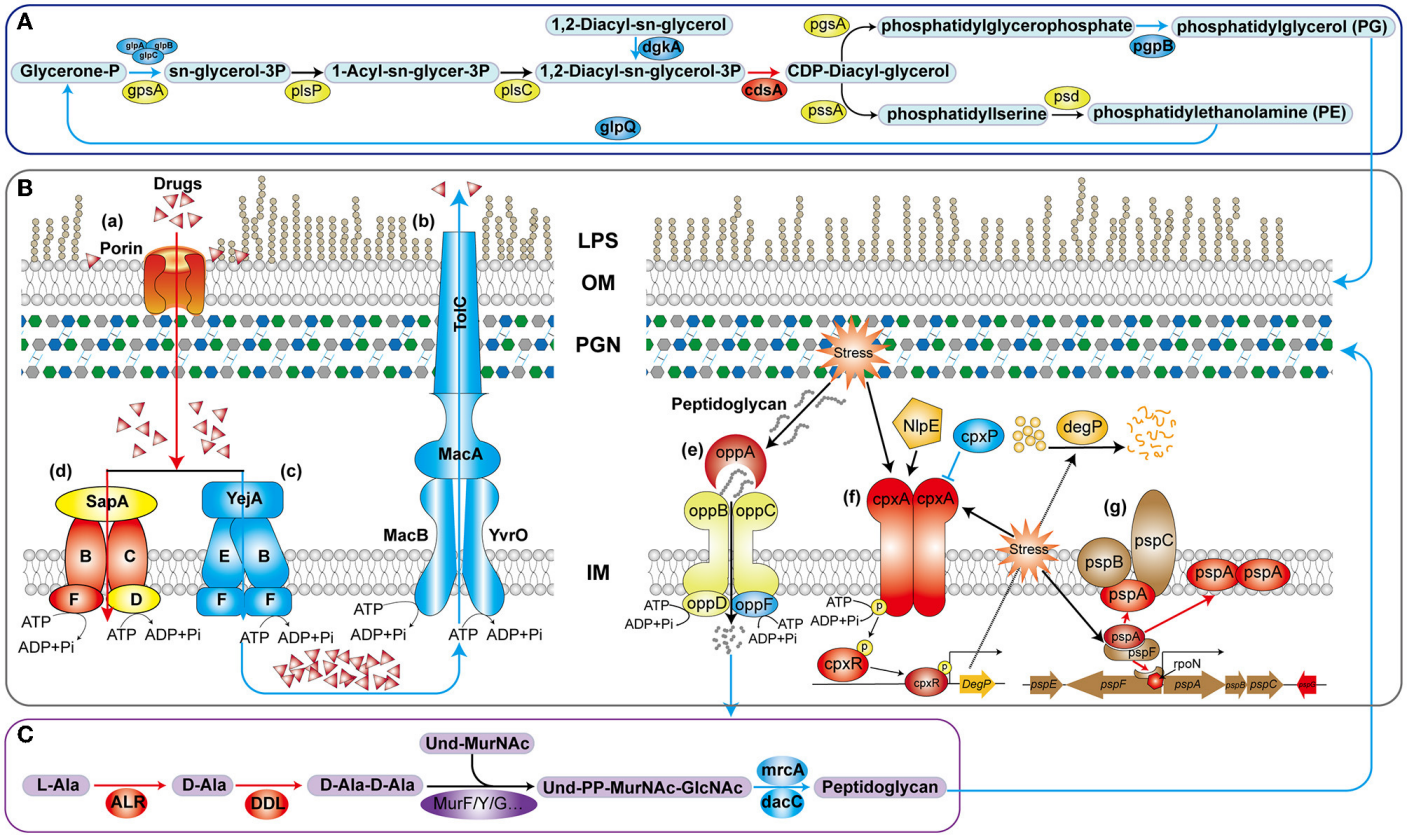
Schematic diagram of the mechanism of *htpG*-mediated antimicrobial peptide and β-lactam antibiotics resistance in *V. mimicus*. The blue lines and color patches represent down-regulated processes or genes corresponding to proteins, while the red ones represent up-regulated ones. **(A)** Key pathways in glycerophospholipid biosynthesis. **(B)** Drug-resistant membrane proteins (a–d) and metabolic mechanism peptidoglycan (e–g). LPS, lipopolysaccharide; OM, outer membrane; PGN, peptidoglycan; IM, inner membrane. **(C)** Key reactions in peptidoglycan synthesis for bacterial cell wall.

Gram-negative bacteria have a multi-layered envelope consisting of an outer membrane (OM), inner membrane (IM), and peptidoglycan (PGN) layer. Both the outer and inner membranes feature a phospholipid bilayer structure rich in proteins, while the OM is additionally adorned with lipopolysaccharides (LPS). LPS constitute a pivotal component of the OM in the majority of Gram-negative bacteria, playing a crucial role in safeguarding bacteria from environmental stressors and contributing to antibiotic resistance and pathogenesis (Di Lorenzo et al., 2022). Polymyxin B disrupts the stability of the OM by targeting phospholipids and, with higher affinity, the lipid A domain of LPS (Ledger et al., 2022). In *V. mimicus*, the deletion of *htpG* resulted in a significant upregulation of four genes involved in LPS biosynthesis and assembly pathways. In particular, LapA and LapB serve as checkpoints for the proper assembly of LPS, ensuring that only fully synthesized LPS are delivered to the Lpt complex, a process crucial for bacterial growth. This may result in an increased accumulation of LPS on the bacterial surface, thereby enhancing the binding and lethality of polymyxin B, which aligns with the observed changes in antibiotic resistance phenotypes. In the biosynthesis pathway of glycerophospholipids (Figure 8A), the enzyme complex GlpA/B/C is responsible for converting dihydroxyacetone phosphate into glycerol-3-phosphate, which serves as a precursor for phospholipid synthesis. On the other hand, GlpQ, which functions as a periplasmic glycerophosphoryl diester phosphodiesterase, has the ability to hydrolyze deacylated phospholipids into glycerol-3-phosphate and alcohol. This strategy involves bacteria modifying the surface properties of phospholipids as a means to evade antimicrobial peptides. In the final step, PgpB synthesizes phosphatidylglycerol (PG) from its precursor, phosphatidylglycerol phosphate (PGP). Additionally, because of its extensive substrate connectivity, PgpB plays a crucial role in linking the biosynthesis of membrane glycerophospholipids and cell wall polysaccharides. The significant downregulation of these five genes may inhibit glycerophospholipid synthesis, disrupt the balance of its production, and consequently affect membrane fluidity, permeability, and stability. In *Staphylococcus aureus*, reduced phosphatidylglycerol content results in a more a more negative net charge, thereby enhancing the binding of cationic antimicrobial peptides and increasing sensitivity (Nishi et al., 2004), which is consistent with the phenotypic changes observed in this study.

In the drug-resistant membrane proteins, porins exhibited an overall upregulation (Figure 8B-a). The contribution of *ompU, ompA, ompW*, and *lamB* genes to the susceptibility of several β-lactams has been confirmed in pathogenic bacteria such as *V. cholerae, Acinetobacter baumannii, Salmonella enterica*, and *Klebsiella pneumoniae*, respectively (Nguyen et al., 2009; Garcia-Sureda et al., 2011; Smani et al., 2014; Ko and Choi, 2022). Surprisingly, despite the upregulation of porin expression due to *htpG* deletion in *V. mimicus*, its sensitivity to β-lactams decreased, suggesting that porins may not be the primary targets for β-lactams resistance. Besides drug resistance, porins also serve as essential nutrient uptake channels in bacteria, regulated by responsive regulatory elements coordinating with stress pathways (Fernández and Hancock, 2013). Therefore, the increased porin expression might represent an adaptive response to unfavorable conditions caused by the absence of the vital molecular chaperone protein HtpG. All five genes within the MacAB-TolC efflux pump displayed significant downregulation. MacAB, in conjunction with the OM protein TolC, creates a tripartite channel that facilitates the efflux of a diverse range of antimicrobial compounds, as well as endogenous molecules and toxins (Figure 8B-b). Studies conducted on *E. coli* and clinical isolates of several other bacteria have substantiated that overexpression of MacA and MacB proteins imparts resistance to macrolide antibiotics and antimicrobial peptides like polymyxin B (Greene et al., 2018). This study also observed a significant decrease in resistance to azithromycin (AZM) and polymyxin B (PMB) in the Δ*htpG* strain (Figure 2B-a), which is consistent with the findings of these two studies, indicating the significant role of the MacAB-TolC efflux pump in the *htpG*-mediated regulation of antimicrobial resistance in *V. mimicus*. Two high-affinity peptide ABC transport systems, YejABEF and SapABCDF, have been confirmed to confer resistance to antimicrobial peptides in bacteria. The potential mechanism involves transferring antimicrobial peptides into the cytoplasm, thereby keeping them away from their targets and exposing them to intracellular degradation (Figure 8B-c, d; Garai et al., 2017). Studies in *Salmonella enterica* and *S. typhimurium* have also shown that enhancing the function of YejABEF and SapABCDF systems increases resistance to antimicrobial peptides (Groisman et al., 1992; Eswarappa et al., 2008). However, upon deleting the *htpG*, these two functionally similar systems exhibit completely opposite gene expression patterns. Considering the decreased resistance of the Δ*htpG* strain to polymyxin B, we speculate that the overall downregulation of the YejABEF pump plays a predominant role, while the compensatory upregulation of the SapABCDF pump counterbalances its physiological impact. Further investigation into the regulatory interactions and the order of significance of these systems will contribute to a clearer understanding of specific targets for the development of strategies to reduce polymyxin resistance.

In addition to the cell membrane, the cell wall, mainly composed of peptidoglycan, is also an crucial component of the bacterial envelope. Alanine racemase (Alr) and d-Alanine-d-alanine ligase (Ddl) are the key enzymes in the D-Ala-D-Ala branch of peptidoglycan biosynthesis (Qin et al., 2021; Figure 8C) and are an important drug discovery target. Alr is responsible for supplying d-alanine, while Ddl facilitates the condensation of two alanine molecules utilizing ATP, which serves as the terminal peptide in peptidoglycan monomer formation. On the other hand, PBPs regulates the assembly of bacterial cell wall peptidoglycan layers, and are targets of β-lactams. The MrcA (PBP1A) and dacC (PBP5) are major peptidoglycan synthases (Kang and Boll, 2022) and D-alanyl-D-alanine carboxypeptidases, respectively, located at the end of the peptidoglycan biosynthetic pathway. In the Δ*htpG* strain, enzymes responsible for synthesizing peptidoglycan precursors were upregulated, while those involved in synthesizing the final product were downregulated. Consequently, it can be inferred that the reduction in the final product, peptidoglycan, severely impacts cell wall synthesis and structure. Simultaneously, the decrease in PBPs would lead to a reduction in β-lactam targets, thereby enhancing resistance correspondingly. This is consistent with the observed phenotypic changes in this study. The upregulation of *ddl* and *alr* may be attributed to the presence of negative feedback regulation within the peptidoglycan synthesis pathway. Furthermore, peptidoglycan is a sugar-amino acid polymer, possesses high immunogenicity. To conserve energy and evade host detection, bacteria recycle peptidoglycan by transporting it into the cytoplasm for new synthesis (Singh et al., 2020). The bacterial oligopeptide permeases (opp) system, a membrane-associated complex comprising five proteins of the ABC transport family (Figure 8B-e), plays a crucial role in peptidoglycan and cell wall peptide recycling in Gram-negative bacteria. Upon deleting the *htpG*, the substrate-binding protein VM_09400 (*oppA*) exhibited significant upregulation, indicating that the bacterium reinforces the recycling of cell wall peptides and peptidoglycan by increasing *oppA* production. However, the ATP-binding protein VM_19555 (*oppF*), responsible for energy supply, showed significant downregulation, indicating insufficient energy for the system and a potential failure in the recycling of cell wall peptides and peptidoglycan.

Owing to the vital role of the cell envelope, prokaryotes have developed diverse mechanisms to meticulously oversee and preserve the integrity of their cell envelope. Two such systems are the CpxA/CpxR two-component system and the phage shock protein (psp) system. The Cpx system can regulate the function of some peptidoglycan amidases or AcrAB-TolC efflux pumps, thereby modifying bacterial resistance to some β-lactam and cationic antimicrobial peptide antibiotics. This system includes the pressure-sensing kinase CpxA located in the IM, the response regulator CpxR in the cytoplasm, and the auxiliary periplasmic protein CpxP, which inhibits CpxA through a direct dynamic interaction (Figure 8B-f). Normal activation of The Cpx system boosts chaperone and protease expression, along with enhancing peptidoglycan modifications by proteins, all contributing to the response of cell to envelope stress (Mitchell and Silhavy, 2019). In the process of activation and response, two of the famous proteins are NlpE, an OM lipoprotein that acts as a signaling module activator (Cho et al., 2023), and DegP, a periplasmic protease triggered to break down misfolded proteins upon CpxA/CpxR system activation. However, this study observed that the gene expression levels of *nlpE* (VM_05160) and *degP* (VM_12340) were downregulated, although not significantly. It suggests that response of the Cpx system to envelope stress may be attenuated. Further investigation could be needed to understand the specific factors or mechanisms responsible for this observation and its implications for bacterial stress responses and antibiotic resistance. In another psp system (Figure 8B-g), under non-stress conditions, pspA inhibits the activity of the transcription factor pspF. However, in response to envelope stress, pspA dissociates from pspF, allowing pspF to activate RNA polymerase factor sigma-54 (RpoN) dependent transcription of the psp operon. Furthermore, pspA not only associates with the membrane-bound pspBC to maintain membrane stability but also directly interfaces with the cell membrane, ensuring its stability. Consequently, pspA serves as a regulatory element, sensor module, and effector protein, highlighting its central role within the psp network (Popp et al., 2022). In Δ*htpG* strain, only the core genes *pspA*, functionally uncharacterized *pspG*, and the transcriptional regulator *rpoN* were significantly upregulated, while other gene changes were not significant. This suggests that the bacteria are responding to the envelope stress triggered by *htpG* deletion to adapt to external pressures, but downstream genes are not transcriptionally regulated in response, indicating the presence of other regulatory mechanisms or unobserved responses.

## 5 Conclusions

In conclusion, the increased sensitivity of Δ*htpG* strain to antimicrobial peptides can be attributed to multiple factors: (1) upregulation of LPS synthesis, providing more binding targets; (2) reduction in glycerophospholipid content, promoting charge interactions between drugs and bacterial cells; (3) reduced efflux activity of the MacAB-TolC pump, leading to increased drug retention within cells; (4) weakened inward transport and digestion of the YejABEF pump, allowing more drugs to remain in their active forms. Meanwhile, the decreased sensitivity of the Δ*htpG* strain to β-lactam antibiotics can be attributed to: (1) reduced peptidoglycan synthesis, resulting in fewer PBPs and targets; (2) dysregulation of peptidoglycan recycling and envelope stress response. Further exploration of specific pathway components is essential for a comprehensive understanding of *htpG*-mediated resistance mechanisms, aiding in antimicrobial agent development.

## Data availability statement

The datasets presented in this study can be found in online repositories. The names of the repository/repositories and accession number(s) can be found at: https://bigd.big.ac.cn/gsa/browse/CRA009391.

## Author contributions

ZQ: Conceptualization, Data curation, Formal analysis, Investigation, Methodology, Project administration, Visualization, Writing—original draft, Writing—review & editing. KP: Data curation, Investigation, Methodology, Visualization, Writing—review & editing. YF: Writing—review & editing. YW: Software, Visualization, Writing—review & editing. BH: Data curation, Formal analysis, Methodology, Writing—review & editing. ZT: Methodology, Validation, Writing—review & editing. PO: Project administration, Resources, Writing—review & editing. XH: Funding acquisition, Project administration, Resources, Writing—review & editing. DC: Project administration, Resources, Writing—review & editing. WL: Project administration, Resources, Writing—review & editing. YG: Data curation, Funding acquisition, Resources, Supervision, Writing—review & editing.

## Footnotes

^1^ https://github.com/OpenGene/fastp

^2^ http://www.geneontology.org/

^3^ http://www.genome.jp/kegg/

^4^ http://deweylab.github.io/RSEM/

^5^ http://bioconductor.org/packages/stats/bioc/DESeq2/

^6^ https://www.majorbio.com

## Supplementary material

Supplementary Figure 1 DEGs volcano plot and PCA analysis of transcriptional profiles of *V. mimicus* WT and Δ*htpG* strains. **(A)** The volcano plot of DEGs between *V. mimicus* WT and Δ*htpG* strains, including mRNA and sRNA. Red dots represent up-regulated DEGs, blue dots represent down-regulated DEGs, and gray dots represent no differentially expressed genes. **(B)** Principal Component Analysis (PCA) based on sample expression levels is performed (the x and y-axis represent the calculated values of the principal components, with each point representing a different sample. The distance between samples indicates their similarity, and the ellipses denote the 95% confidence interval).

Supplementary Table 1 Primers used for RT-qPCR analysis.

Supplementary Table 2 List of antibacterial agents and their targets.

Supplementary Table 3 Biochemical phenotype of test strains.

Supplementary Table 4 Partial annotation and expression level of all DEGs.

Supplementary Table 5 Go enrichment analysis of up-regulated mRNA.

Supplementary Table 6 KEGG enrichment analysis of all DEGs.
