# Corrigendum: Transcriptome reveals the role of the *htpG* gene in mediating antibiotic resistance through cell envelope modulation in *Vibrio mimicus* SCCF01

**DOI:** 10.3389/fmicb.2024.1381070

**Published:** 2024-03-13

**Authors:** Zhenyang Qin, Kun Peng, Yang Feng, Yilin Wang, Bowen Huang, Ziqi Tian, Ping Ouyang, Xiaoli Huang, Defang Chen, Weimin Lai, Yi Geng

**Affiliations:** ^1^College of Veterinary Medicine, Sichuan Agricultural University, Chengdu, Sichuan, China; ^2^Department of Aquaculture, Sichuan Agricultural University, Chengdu, Sichuan, China

**Keywords:** HtpG, *Vibrio mimicus*, transcriptome, drug resistance, cell wall, cell membrane

In the published article, there was an error in the **Funding** statement, page 13.

The previous Funding statement:

“The author(s) declare financial support was received for the research, authorship, and/or publication of this article. This research was supported by National Natural Science Foundation of China (32373174), Sichuan Natural Science Foundation (2024YJ2011), and Sichuan Innovation Team Project of Agricultural Industry Technology System (SCCXTD-15).

The correct Funding statement appears below:

The author(s) declare financial support was received for the research, authorship, and/or publication of this article. This research was supported by National Natural Science Foundation of China (32373174), Sichuan Natural Science Foundation (24NSFSC0858), and Sichuan Innovation Team Project of Agricultural Industry Technology System (SCCXTD-15).

The authors apologize for this error and state that this does not change the scientific conclusions of the article in any way. The original article has been updated.

