# Supplementary figures and images for: Transcriptome reveals the role of the htpG gene in mediating antibiotic resistance through cell envelope modulation in Vibrio mimicus SCCF01
Source: Front Microbiol. 2024 Jan 4;14:1295065. doi: 10.3389/fmicb.2023.1295065 (PMC10794384; doi:10.3389/fmicb.2023.1295065)

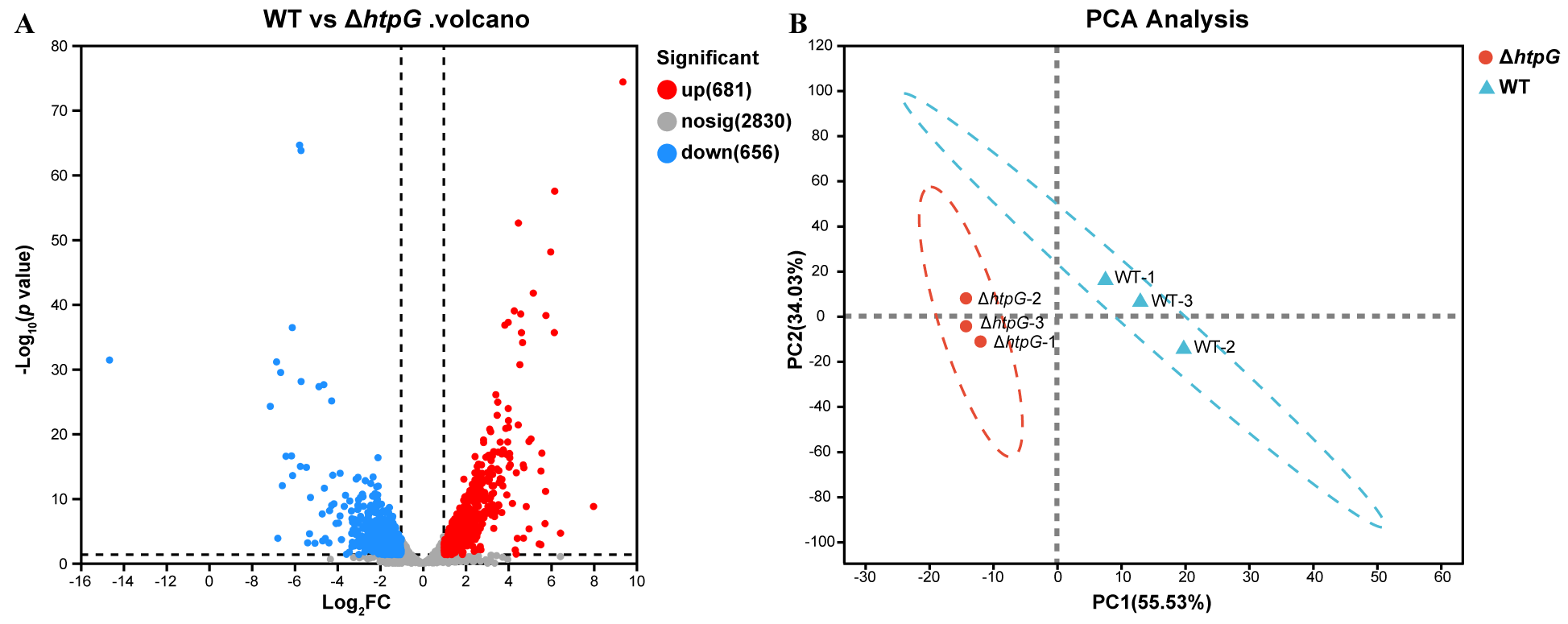

Supplement: Supplementary Figure 1 — DEGs volcano plot and PCA analysis of transcriptional profiles of V. mimicus WT and ΔhtpG strains. (A) The volcano plot of DEGs between V. mimicus WT and ΔhtpG strains, including mRNA and sRNA. Red dots represent up-regulated DEGs, blue dots represent down-regulated DEGs, and gray dots represent no differentially expressed genes. (B) Principal Component Analysis (PCA) based on sample expression levels is performed (the x and y-axis represent the calculated values of the principal components, with each point representing a different sample. The distance between samples indicates their similarity, and the ellipses denote the 95% confidence interval). [file Image_1.TIF]
